# Curcumin is a potent modulator of microglial gene expression and migration

**DOI:** 10.1186/1742-2094-8-125

**Published:** 2011-09-29

**Authors:** Marcus Karlstetter, Elena Lippe, Yana Walczak, Christoph Moehle, Alexander Aslanidis, Myriam Mirza, Thomas Langmann

**Affiliations:** 1 Institute of Human Genetics, University of Regensburg, Franz-Josef-Strauss-Allee 11, 93053 Regensburg, Germany; 2 Center of Excellence for Fluorescent Bioanalytics, University of Regensburg, Josef-Engert-Str. 9, 93053 Regensburg, Germany

## Abstract

**Background:**

Microglial cells are important effectors of the neuronal innate immune system with a major role in chronic neurodegenerative diseases. Curcumin, a major component of tumeric, alleviates pro-inflammatory activities of these cells by inhibiting nuclear factor kappa B (NFkB) signaling. To study the immuno-modulatory effects of curcumin on a transcriptomic level, DNA-microarray analyses were performed with resting and LPS-challenged microglial cells after short-term treatment with curcumin.

**Methods:**

Resting and LPS-activated BV-2 cells were stimulated with curcumin and genome-wide mRNA expression patterns were determined using DNA-microarrays. Selected qRT-PCR analyses were performed to confirm newly identified curcumin-regulated genes. The migration potential of microglial cells was determined with wound healing assays and transwell migration assays. Microglial neurotoxicity was estimated by morphological analyses and quantification of caspase 3/7 levels in 661W photoreceptors cultured in the presence of microglia-conditioned medium.

**Results:**

Curcumin treatment markedly changed the microglial transcriptome with 49 differentially expressed transcripts in a combined analysis of resting and activated microglial cells. Curcumin effectively triggered anti-inflammatory signals as shown by induced expression of *Interleukin 4 *and *Peroxisome proliferator activated receptor α*. Several novel curcumin-induced genes including *Netrin G1*, *Delta-like 1*, *Platelet endothelial cell adhesion molecule 1*, and *Plasma cell endoplasmic reticulum protein 1*, have been previously associated with adhesion and cell migration. Consequently, curcumin treatment significantly inhibited basal and activation-induced migration of BV-2 microglia. Curcumin also potently blocked gene expression related to pro-inflammatory activation of resting cells including *Toll-like receptor 2 *and *Prostaglandin-endoperoxide synthase 2*. Moreover, transcription of *NO synthase 2 *and *Signal transducer and activator of transcription 1 *was reduced in LPS-triggered microglia. These transcriptional changes in curcumin-treated LPS-primed microglia also lead to decreased neurotoxicity with reduced apoptosis of 661W photoreceptor cultures.

**Conclusions:**

Collectively, our results suggest that curcumin is a potent modulator of the microglial transcriptome. Curcumin attenuates microglial migration and triggers a phenotype with anti-inflammatory and neuroprotective properties. Thus, curcumin could be a nutraceutical compound to develop immuno-modulatory and neuroprotective therapies for the treatment of various neurodegenerative disorders.

## Background

Microglial cells are resident macrophages of the nervous system with pivotal roles in innate immune regulation and neuronal homeostasis [1,2]. They are cells of the mononuclear phagocyte lineage but their unique localization within the nervous system and their morphological features clearly distinguish them from other macrophage populations [3]. Ramified microglial cells actively scan their environment with their long protrusions [4,5] and continuous inhibitory signals from neurons prevent microglial toxicity [6,7]. Disconnection of the microglia-neuron cross-talk [8], local danger signals such as released ATP [9], or neurotransmitter gradients [10] can lead to a functional transformation of microglial populations with a variety of effector functions. Consequently, alarmed microglia and reactive microgliosis have been identified in a variety of neurodegenerative diseases including Alzheimer's disease [11], Parkinson's disease [12], amyotrophic lateral sclerosis [13], multiple sclerosis [14], and inherited photoreceptor dystrophies [15]. The concept of a microglia-targeted pharmacotherapy to prevent neurodegeneration in the brain and the retina is therefore a promising approach under active investigation [16,17].

There is a growing interest in the identification of natural compounds that limit neuroinflammation and simultaneously support neuronal survival [18,19]. Among the naturally occuring immuno-modulators, curcumin ((E, E)-1,7-bis(4-hydroxy-3-methoxyphenyl)-1,6-heptadiene-3,5-dione), a major constituent of tumeric, is a herbal medicine used for centuries in India and China [20]. Curcumin has a wide range of pharmacological activities including anti-inflammatory, anti-microbial, antioxidant, and anti-tumor effects [21]. Curcumin is a particularly potent immuno-regulatory agent that can modulate the activation and function of T-cells, B-cells, neutrophils, natural killer cells and macrophages [22].

Curcumin treatment effectively inhibits the activation of microglial cells by diminishing the production of nitric oxide [23] and reducing the secretion of pro-inflammatory cytokines such as IL1β, IL6 and TNF [24]. Moreover, curcumin blocks the LPS-mediated induction of cyclooxygenase-2 (COX2) via inhibition of the transcription factors nuclear factor kappa B (NFkB), activator protein 1 (AP1), and signal transducers and activators of transcription (STATs) [25,26]. Recent experiments have also demonstrated that curcumin protects dopaminergic neurons against microglia-mediated neurotoxicity [27], limits brain inflammation [28], and rescues retinal cells from stress-induced cell death [29].

The inhibitory role of curcumin on pro-inflammatory gene expression in microglia is well documented. However, this information is limited to only a few well-studied examples including pro-inflammatory cytokines, Nos2 and COX2. In a genome-wide search for target genes, we investigated the transcriptomic effects of curcumin in resting and LPS-activated BV-2 microglial cultures using DNA-microarrays. Furthermore, we validated the curcumin-regulated expression of microglial transcripts with qRT-PCR and studied the related microglial migration and neurotoxicity.

## Methods

### Reagents

Curcumin and *E.coli *0111:B4 lipopolysaccharide were purchased from Sigma Aldrich (Steinheim, Germany). Curcumin was dissolved in DMSO and added in concentrations that did not exceed 0.05% of the total volume in any of the cell culture experiments.

### Cell culture

BV-2 microglia-like cells were provided by Professor Ralph Lucius (Clinic of Neurology, Christian Albrechts University, Kiel, Germany). BV-2 cells were cultured in RPMI/5% FCS supplemented with 2 mM L-Glutamine and 195 nM β-mercaptoethanol. BV-2 cells were stimulated with 100 ng/ml LPS, 20 μM of curcumin, or DMSO as control for 6 h. These stimulation conditions were adapted from previously published experiments [24,30]. MTT assays revealed that 100 ng/ml LPS, 20 μM curcumin, or a combination of both had no cytotoxic effects on BV-2 cells (data not shown). 661W photoreceptor-like cells were a gift from Prof. Muayyad Al-Ubaidi (University of Illinois, Chicago, IL) and the culture conditions have been described elsewhere [31].

### Scratch assay

500.000 BV-2 cells were grown in 6-well plates as 80% confluent monolayers and were wounded with a sterile 100 μl pipette tip. Thereafter, the cells were stimulated with 100 ng/ml LPS, 20 μM of curcumin, 100 ng/ml LPS + 20 μM of curcumin, or DMSO as solvent control. Migration into the open scar was documented with microphotographs at different time points after wounding. The number of migrating cells was quantified by counting all cells within a 0.4 mm^2 ^region in the center of each scratch. A minimum of 5 individual cultures was used to calculate the mean migratory capacity of each cell culture condition.

### Transwell migration assay

The Costar Transwell System (8-μm pore size polycarbonate membrane) was used to evaluate vertical cell migration. 1 Mio BV-2 cells in 1.5 ml serum-free medium were added to the upper well, and 2.6 ml serum-free medium was added to the lower chamber. 100 ng/ml LPS, 20 μm curcumin, 100 ng/ml LPS + 20 μm curcumin, or DMSO as solvent control were added to the lower chamber medium. At the end of a 24 h incubation period, cells that had migrated to the lower surface were quantified by counting the migrated cells on the lower surface of the membrane using microscopy.

### 661W co-culture in microglia-conditioned medium and apoptosis assay

To test microglial neurotoxicity, a culture system of 661W photoreceptors with microglia conditioned medium was established. 661W cells were incubated for 48 h either in their own medium or with culture supernatants from unstimulated, 100 ng/ml LPS, 20 μM curcumin, or 100 ng/ml LPS + 20 μM curcumin treated microglial cells. The 661W cell morphology was assessed by phase contrast microscopy and apoptotic cell death was determined with the Caspase-Glo^® ^3/7 Assay (Promega). Cells were lysed and incubated with a luminogenic caspase-3/7 substrate, which contains the tetrapeptide sequence DEVD. Luminescence was then generated by addition of recombinant luciferase and was proportional to the amount of caspase activity present. The luminescent signal was read on a BMG FluoStar Optima plate reader (Labtech, Offenburg, Germany). A blank reaction was used to measure background luminescence associated with the cell culture system and Caspase-Glo^® ^3/7 Reagent. The value for the blank reaction was subtracted from all experimental values. Negative control reactions were performed to determine the basal caspase activity of 661W cells. Relative luciferase units (RLU) reflect the level of apoptotic cell death in the different 661W cell cultures.

### RNA isolation and reverse transcription

Total RNA was extracted from cultured microglial cells according to the manufacturer's instructions using the RNeasy Protect Mini Kit (Qiagen, Hilden, Germany). Purity and integrity of the RNA was assessed on the Agilent 2100 bioanalyzer with the RNA 6000 Nano LabChip^® ^reagent set (Agilent Technologies, Böblingen, Germany). The RNA was quantified spectrophotometrically and then stored at -80°C. First-strand cDNA synthesis was performed with RevertAid™ H Minus First Strand cDNA Synthesis Kit (Fermentas, St. Leon-Rot, Germany).

### DNA-microarray analysis

4 × 44 K microarrays (014868) (Agilent Technologies) were used for hybridization with three independent RNAs from non-stimulated BV-2 microglial cells or cultures treated for 6 h with 20 μM curcumin, 100 ng/ml LPS, or 20 μM curcumin + 100 ng/ml LPS, respectively. Briefly, 200 ng of total RNA were labeled with Cy3 using the Agilent Quick-Amp Labeling Kit - 1 color according to the manufacturer's instructions. cRNA was purified with the RNeasy Mini Kit (Qiagen) and labeling efficiency was determined with a NanoDrop ND-1000 photometer (PeqLab). The arrays were incubated with cRNAs in Agilent SureHyb chambers for 17 hours at 65°C while rotating. After washing, scanning was done with the Agilent G2565CA Microarray Scanner System and the resulting TIFF files were processed with Agilent Feature Extraction software (10.7.). Minimum information about a microarray experiment (MIAME) criteria were met [32]. The microarray dataset of this study is publicly available at the National Center for Biotechnology Information Gene Expression Omnibus http://www.ncbi.nlm.nih.gov/geo/ as series record GSE23639.

### Bioinformatic data analysis

Integrative analysis of genome-wide expression activities from BV-2 cells was performed with the Gene Expression Dynamics Inspector (GEDI), a Matlab (Mathworks, Natick, MA) freeware program which uses self-organizing maps (SOMs) to translate high-dimensional data into a 2D mosaic [33]. Each tile of the mosaic represents an individual SOM cluster and is color-coded to represent high or low expression of the cluster's genes, thus identifying the underlying pattern. The Partek Genomics Suite (Partek Inc.) was used for ANOVA analysis and hierarchical clustering of normalized expression values. Differentially regulated transcrips in curcumin-stimulated versus non-treated and curcumin + LPS versus LPS-treated BV-2 cells, respectively, were retrieved with the Genomatix ChipInspector program (Genomatix Software GmbH, Munich, Germany), applying the Significance Analysis of Microarray (SAM) algorithm using a false-discovery rate of 0.1%.

### Quantitative real-time RT-PCR

Amplifications of 50 ng cDNA were performed with an ABI7900HT machine (Applied Biosystems) in triplicates in 10 μl reaction mixtures containing 1×TaqMan Universal PCR Master Mix (Applied Biosystems), 200 nM of primers and 0.25 μl dual-labeled probe (Roche ProbeLibrary). The reaction parameters were as follows: 2-min 50°C hold, 30-min 60°C hold, and 5-min 95°C hold, followed by 45 cycles of 20-s 94°C melt and 1-min 60°C anneal/extension. Measurements were performed in triplicate. Results were analyzed with an ABI sequence detector software version 2.3 using the ΔΔCt method for relative quantitation. A Ct (cycle threshold) < 35 was used as cutoff for estimating significantly expressed transcripts and cDNA samples with values > 35 were marked with n.e. for not expressed. Ct-values between 35 and 40 were solely used for calculation of relative expression differences in treated cells versus control cells. Primer sequences and Roche Library Probe numbers are listed in Table 1.

**Table 1:** Primer pairs and Roche library probes for real-time qRT-PCR validation

Gene	F-Primer (5'-3')	R-Primer (5'-3')	Roche Library Probe
*Atp5b*	ggcacaatgcaggaaagg	tcagcaggcacatagatagcc	77
*C3*	accttacctcggcaagtttct	ttgtagagctgctggtcagg	76
*Ccl2*	catccacgtgttggctca	gatcatcttgctggtgaatgagt	62
*Dll1*	ttcaactgtgagaagaagatggat	gccgaggtccacacactt	103
*Egr2*	ctacccggtggaagacctc	aatgttgatcatgccatctcc	60
*Il4*	catcggcattttgaacgag	cgagctcactctctgtggtg	2
*Il6*	gatggatgctaccaaactggat	ccaggtagctatggtactccaga	6
*Nos2*	ctttgccacggacgagac	tcattgtactctgagggctga	13
*Ntng1*	aggggcaagagaccaagg	agggatggtgtctatcgtcct	103
*Pecam1*	cggtgttcagcgagatcc	cgacaggatggaaatcacaa	45
*Perp1*	tcatatgccggctcacct	atccactggcgtctggagt	110
*Pparα*	ccgagggctctgtcatca	gggcagctgactgaggaa	11
*Ptgs2*	gatgctcttccgagctgtg	ggattggaacagcaaggattt	45
*Stat1*	aaatgtgaaggatcaagtcatgtg	catcttgtaattcttctagggtcttga	15
*Tlr2*	accgaaacctcagacaaagc	cagcgtttgctgaagagga	49

### Statistical analyses

Statistical analyses were performed on ΔΔCt data using the Mann-Whitney Rank Sum test and quantitative expression data are expressed as mean ± SD plotted at a logarithmic scale. Gene expression levels in control BV-2 cells were used as calibrators. The Student's t test or Mann-Whitney Rank Sum test were used for the comparison of experimental groups in cell migration assays and apoptosis assays as indicated. p < 0.05 was considered significant.

## Results

### Curcumin has a major impact on the microglial transcriptome

**Figure 1:**
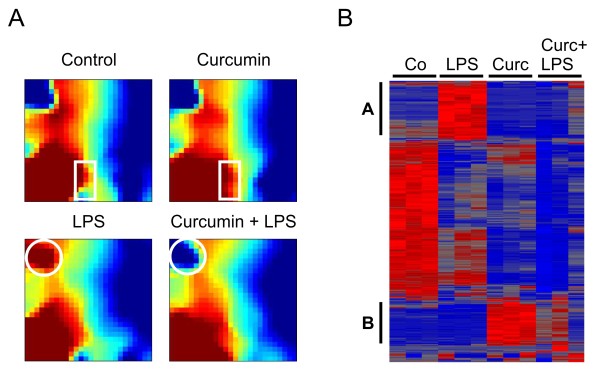
**Curcumin influences global gene expression in resting and LPS-activated BV-2 microglial cells**. (A) Gene Expression Dynamics Inspector (GEDI) analysis of the complete DNA-microarray dataset from control BV-2 cells or cells treated with 20 μM curcumin, 100 ng/ml LPS, or 20 μM curcumin + 100 ng/ml LPS for 6 hours. The white rectangles and circles denote the most prominent expression changes in corresponding gene clusters. (B) Hierarchical clustering of normalized expression levels after one-way ANOVA at p < 0.01. Triplicate microarrays were analyzed for each condition and the pseudo-color scale indicates high (red) or low (blue) expression levels. LPS and curcumin-related clusters are marked with A and B, respectively.

To determine the transcriptional profiles of resting and LPS-activated BV-2 microglial cells after treatment with 20 μM curcumin for 6 h, we performed DNA-microarray analyses from three independent stimulations. We first applied the Gene Expression Dynamics Inspector (GEDI) on the complete dataset to visualize the global patterns of gene expression in the four different conditions, untreated, curcumin-treated, LPS-treated, and curcumin + LPS-treated cells. GEDI uses self-organizing maps to capture genome-wide transcriptome activity via 'gestalt' recognition [33]. GEDI facilitates the identification of genome-wide patterns with each mosaic tile in the map representing a gene cluster that is expressed at similar levels. The four GEDI maps, with blue color indicating low and red color high mRNA expression levels, show a dynamic regulation of gene transcription in the cultured microglial cells (Figure 1A). The major difference between curcumin-treated resting microglial cells and control cells was a region with higher expression at the bottom of the map (Figure 1A, white rectangles). In the LPS-treated condition, mimicking a highly activated state, curcumin elicited a largely converse expression pattern with a pronounced area of weakly expressed genes (Figure 1A, white circles). These data indicate that curcumin stimulates gene expression in resting, non-activated cells but mainly dampens activation-associated transcriptional programs in LPS-primed microglia.

We next calculated hierarchical clusters of each individual microarray dataset after filtering for significantly altered gene expression using one way ANOVA at p < 0.01. This analysis showed a clear separation of the four different conditions with their own characteristic gene expression profiles (Figure 1B). The clustering revealed two distinct groups of inversely regulated genes. Group A (Figure 1B) contains LPS-induced genes, which are no longer up-regulated in the presence of curcumin. Group B (Figure 1B) represents genes selectively up-regulated by curcumin treatment of resting microglial cells. Together with the GEDI analysis, these results demonstrate that stimulation with curcumin impacts distinct patterns of gene expression in resting and LPS-activated microglial cells, respectively.

To narrow down the identified global gene clusters to a subset of genes with significantly different mRNA expression in the different curcumin-treated conditions, we used the Genomatix ChipInspector tool applying the Significance Analysis of Microarray (SAM) algorithm at a false discovery rate of 0.1% and a minimum fold change of 2.0 [34]. Thereby, 35 significantly regulated transcripts were identified in curcumin-treated versus resting microglial cells (Table 2) and 30 differentially expressed genes were detected in curcumin + LPS versus LPS-stimulated cells (Table 3). Comparison of the total numer of differentially expressed transcripts and considering overlapping gene sets revealed that curcumin affects both resting and LPS-activated BV-2 cells.

**Table 2:** Differentially expressed transcripts after 6 h stimulation of BV-2 cells with 20 μM curcumin

Nr	ID	Symbol	Gene Name	FC
**UP-REGULATED**				
1	80883	Ntng1	Netrin G1	313.0
2	18613	Pecam1	Platelet/endothelial cell adhesion molecule 1	42.8
3	13388	Dll1	Delta-like 1	35.5
4	12824	Col2a1	Collagen, type II, alpha 1	21.6
5	14103	Fasl	Fas ligand (TNF superfamily, member 6)	12.8
6	12653	Chgb	Chromogranin B	11.9
7	66184	Rps4y2	Ribosomal protein S4, Y-linked 2	9.7
8	69816	Perp1	RIKEN cDNA 2010001M09 gene	9.7
9	16992	Lta	Lymphotoxin A	9.4
10	19206	Ptch1	Patched homolog 1	5.9
11	19013	Ppara	Peroxisome proliferator activated receptor alpha	4.6
12	109685	Hyal3	Hyaluronoglucosaminidase 3	4.5
13	20997	T	Brachyuri	4.2
14	17246	Mdm2	Transformed mouse 3T3 cell double minute 2	3.1
15	14102	Fas	Fas	2.8
16	14183	Fgfr2	Fibroblast growth factor receptor 2	2.7
17	13645	Egf	Epidermal growth factor	2.2
18	14179	Fgf8	Fibroblast growth factor 8	2.1
19	20655	Sod1	Superoxide dismutase 1	2.1
20	14526	Gcg	Glucagon	2.0
**DOWN-REGULATED**				
1	24088	Tlr2	Toll-like receptor 2	-6.9
2	18505	Pax3	Paired box gene 3	-6.6
3	14281	Fos	FBJ osteosarcoma oncogene	-4.4
4	11622	Ahr	Aryl-hydrocarbon receptor	-4.1
5	16835	Ldlr	Low density lipoprotein receptor	-3.8
6	13654	Egr2	Early growth response 2	-3.5
7	68010	Bambi	BMP and activin membrane-bound inhibitor	-3.3
8	12048	Bcl2l1	BCL2-like 1	-3.3
9	19225	Ptgs2	Prostaglandin-endoperoxide synthase 2	-3.0
10	20296	Ccl2	Chemokine (C-C motif) ligand 2	-2.8
11	12393	Runx2	Runt related transcription factor 2	-2.8
12	17311	Kitl	Kit ligand	-2.4
13	16869	Lhx1	LIM homeobox protein 1	-2.4
14	12977	Csf1	Colony stimulating factor 1	-2.4
15	20528	Slc2a4	Solute carrier family 2, member 4	-2.2

Significance analysis of triplicate microarrays was performed with a false discovery rate of 0.1%. ID, Entrez Gene ID; FC: Fold change.

**Table 3:** Differentially expressed transcripts after 6 h stimulation with 20 μM curcumin + 100 ng/ml LPS versus 100 ng/ml LPS

Nr	ID	Symbol	Gene Name	FC
**UP-REGULATED**				
1	80883	Ntng1	Netrin G1	86.2
2	18613	Pecam1	Platelet/endothelial cell adhesion molecule 1	11.7
3	12824	Col2a1	Collagen, type II, alpha 1	9.0
4	12653	Chgb	Chromogranin B	8.1
5	14103	Fasl	Fas ligand (TNF superfamily, member 6)	6.9
6	19206	Ptch1	Patched homolog 1	6.5
7	69816	Perp1	RIKEN cDNA 2010001M09 gene	5.4
8	14526	Gcg	Glucagon	5.0
9	109685	Hyal3	Hyaluronoglucosaminidase 3	4.1
10	16189	Il4	Interleukin 4	3.8
11	20655	Sod1	Superoxide dismutase 1	3.5
12	19013	Ppara	Peroxisome proliferator activated receptor alpha	3.1
13	20997	T	Brachyuri	2.6
14	640627	Gm9789	ENSMUSG00000044227	2.6
15	16147	Ihh	Indian hedgehog homolog	2.6
16	17246	Mdm2	Transformed mouse 3T3 cell double minute 2	2.5
17	99439	Duox1	Dual oxidase 1	2.3
18	257956	Olfr1307	Olfactory receptor 1307	2.3
19	14179	Fgf8	Fibroblast growth factor 8	2.1
**DOWN-REGULATED**				
1	16193	Il6	Interleukin 6	-93.1
2	18126	Nos2	Nitric oxide synthase 2	-55.7
3	19225	Ptgs2	Prostaglandin-endoperoxide synthase 2	-17.6
4	20296	Ccl2	Chemokine (C-C motif) ligand 2	-14.7
5	12266	C3	Complement C3	-9.1
6	20846	Stat1	Signal transducers and activator of transcription 1	-7.4
7	12048	Bcl2l1	BCL2-like 1	-5.4
8	14281	Fos	FBJ osteosarcoma oncogene	-4.6
9	16835	Ldlr	Low density lipoprotein receptor	-4.1
10	16992	Lta	Lymphotoxin A	-3.5
11	17395	Mmp9	Matrix metallopeptidase 9	-2.2

Significance analysis of triplicate microarrays was performed with a false discovery rate of 0.1%. ID, Entrez Gene ID; FC: Fold change.

### qRT-PCR confirmation of novel curcumin target genes in microglial cells

**Figure 2:**
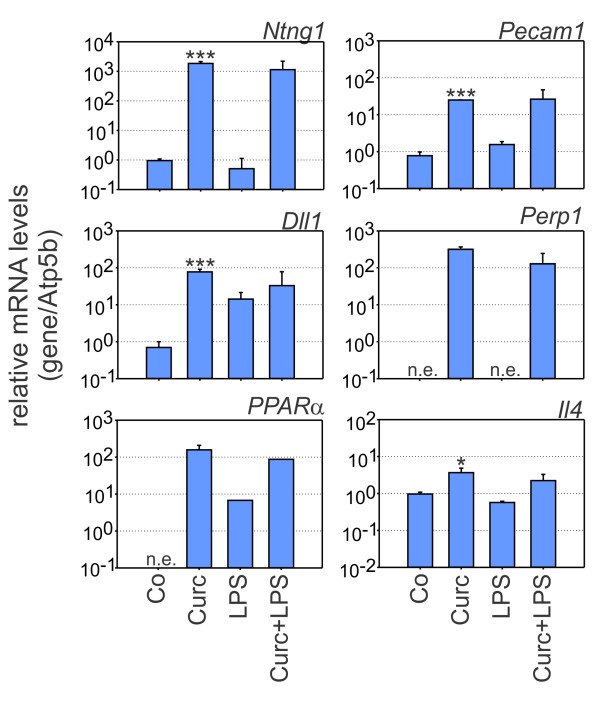
**Curcumin induces genes related to adhesion and anti-inflammatory response**. Real-time qRT-PCR validation of transcripts in BV-2 microglia stimulated with 20 μM curcumin, 100 ng/ml LPS, or 20 μM curcumin + 100 ng/ml LPS for 6 hours. Relative mRNA levels were quantified for Netrin G1 (*Ntng1*), Platelet endothelial call adhesion molecule 1 (*Pecam1*), Delta-like 1 (*Dll1*), Plasma cell induced endoplasmic reticulum protein 1 (*Perp1*), Peroxisome proliferator activated receptor α (*PPARα*), and Interleukin 4 (*Il4*). Expression was normalized to the control gene Atp5b and mRNA levels (+/- SD) are graphed relative to control cells. Results are calculated from three independent experiments performed in triplicate measurements. * p ≤ 0.05, *** p ≤ 0.001 for curcumin vs. control, Mann-Whitney Rank Sum test. n.e., not expressed.

To validate selected differentially expressed genes identified by DNA-microarrays, real-time qRT-PCR assays were performed with RNA samples from three independent BV-2 stimulation series. We especially focused on genes which have not been previously shown to be curcumin targets. In the first set of experiments, mRNA levels of genes highly up-regulated by curcumin compared to control cells were assessed (Figure 2). Transcripts of Netrin G1 (*Ntng1*), Platelet endothelial cell adhesion molecule 1 (*Pecam1*), Delta-like 1 (*Dll1*), Plasma cell endoplasmic reticulum protein 1 (*Perp1*), Peroxisome proliferator activated receptor alpha (*PPARα*), and Interleukin 4 (*Il4*) were all significantly increased by stimulation with curcumin (Figure 2). *Ntng1 *showed the strongest expression difference with a change of more than 1800-fold. *Perp1 *and *PPARα *transcripts were not significantly expressed in resting microglia and were switched on to intermediate levels after curcumin treatment (Figure 2). All six transcripts after combined LPS/curcumin treatment remained similarly high as after curcumin stimulation alone, indicating that the effects of curcumin persist in activated microglial cells.

**Figure 3:**
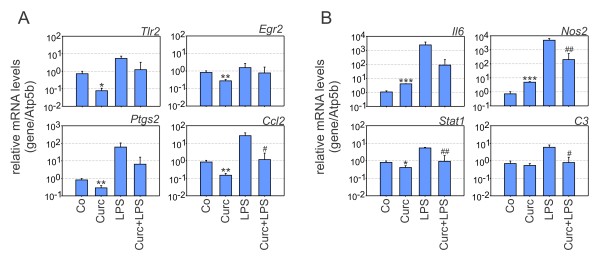
**Curcumin potently blocks pro-inflammatory gene expression**. Real-time qRT-PCR validation of transcripts in BV-2 microglia stimulated with 20 μM curcumin, 100 ng/ml LPS, or 20 μM curcumin + 100 ng/ml LPS for 6 hours. Relative mRNA levels were quantified for (A) Toll-like receptor 2 (*Tlr2*), Early growth response 2 (*Egr2*), Prostaglandin endoperoxide synthase 2 (*Ptgs2*), Chemokine ligand 2 (*Ccl2*), and (B) Interleukin 6 (*Il6*), Nitric oxide synthase 2 (*Nos2*, alias *iNos*), Signal transducer and activator of transcription 1 (*Stat1*), Complement C3 (*C3*). Expression was normalized to the control gene Atp5b and mRNA levels (+/- SD) are graphed relative to control cells. Results are calculated from three independent experiments performed in triplicate measurements. * p ≤ 0.05, ** p ≤ 0.01, *** p ≤ 0.001 for curcumin vs. control, and # p ≤ 0.05, ## p ≤ 0.01 for curcumin + LPS vs. LPS, Mann-Whitney Rank Sum test.

In the next series of qRT-PCR experiments, down-regulated transcripts known to be involved in pro-inflammatory activation of microglial cells were analyzed. Toll-like receptor 2 (*Tlr2*), Early growth response 2 (*Egr2*), Prostaglandin endoperoxide synthase 2 (*Ptgs2*, alias *Cox2*), and Chemokine (C-C-motif) ligand 2 (*Ccl2*, alias *Mcp1*) showed diminished transcript levels in curcumin-treated resting BV-2 cells (Figure 3A). In the activated state, microglial cells also had the tendency to expressed lower amounts of these transcript but only *Ccl2 *levels reached the level of statistical significance. When LPS-activated BV-2 cells were incubated in the presence of curcumin, transcription of Interleukin 6 (*Il6*), nitric oxide synthase 2 (*Nos2*, alias *iNos*), Signal transducer and activator of transcription 1 (*Stat1*), and Complement factor C3 (*C3*) were all repressed (Figure 3B).

### Curcumin has an inhibitory effect on microglial migration

**Figure 4:**
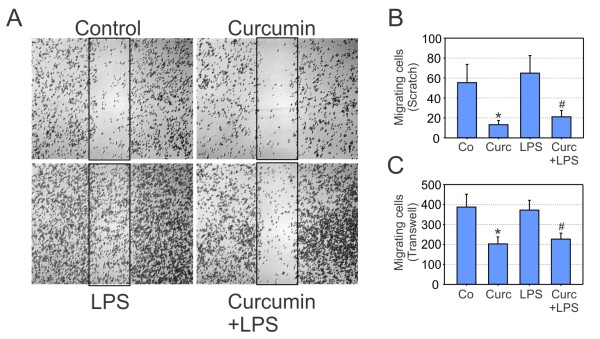
**Curcumin reduces microglial migration**. (A, B) Scratch assays in BV-2 microglia treated with solvent control, 20 μM curcumin, 100 ng/ml LPS, or 20 μM curcumin + 100 ng/ml LPS for 12 hours. (A) Micrographs from one representative experiment out of five independent experiments are shown. (B) Results of scratch assayss are calculated mean values ± SEM from five independent experiments. (C) Transwell chamber migration of BV-2 microglial cells treated with solvent control, 20 μM curcumin, 100 ng/ml LPS, or 20 μM curcumin + 100 ng/ml LPS for 24 hours. The absolute number of migrating cells was counted in the lower chamber and mean values ± SEM are displayed. p ≤ 0.05 for curcumin vs. control, and # p ≤ 0.05 for curcumin + LPS vs. LPS, Student's t test.

The induction of several transcripts related to cell motility and adhesion (*Ntng1*, *Pecam1*, and *Perp1*) prompted us to study the effect of curcumin on microglial migration. We first cultured BV-2 microglia on plastic dishes until 80% confluence and then created a scratch with a pipette tip. 12 hours after stimulation of resting microglia or LPS-activated cells with 20 μM of curcumin, migration into the cell-free scratch area was documented. Representative microscopic images clearly showed that curcumin-treated resting cells as well as activated BV-2 cells exhibit a highly reduced migratory potential (Figure 4A). The statistical analysis of five independent experiments revealed a significantly reduced number of migrating cells when curcumin was present in the culture medium (Figure 4B). As an independent measure of microglial cell motility and to study the long term effects of curcumin, we performed transwell migration assays over a period of 24 hours. Similar as in the scratch assays, the migratory capacity of BV-2 cells was not changed by the activation agent LPS alone (Figure 4C). In both, the resting and the activated microglial phenotype, curcumin caused a significant attenuation of microglial migration (Figure 4C). These results indicate that curcumin-mediated signaling events have functional consequences related to microglial motility.

### Curcumin inhibits LPS-induced microglial neurotoxicity

**Figure 5:**
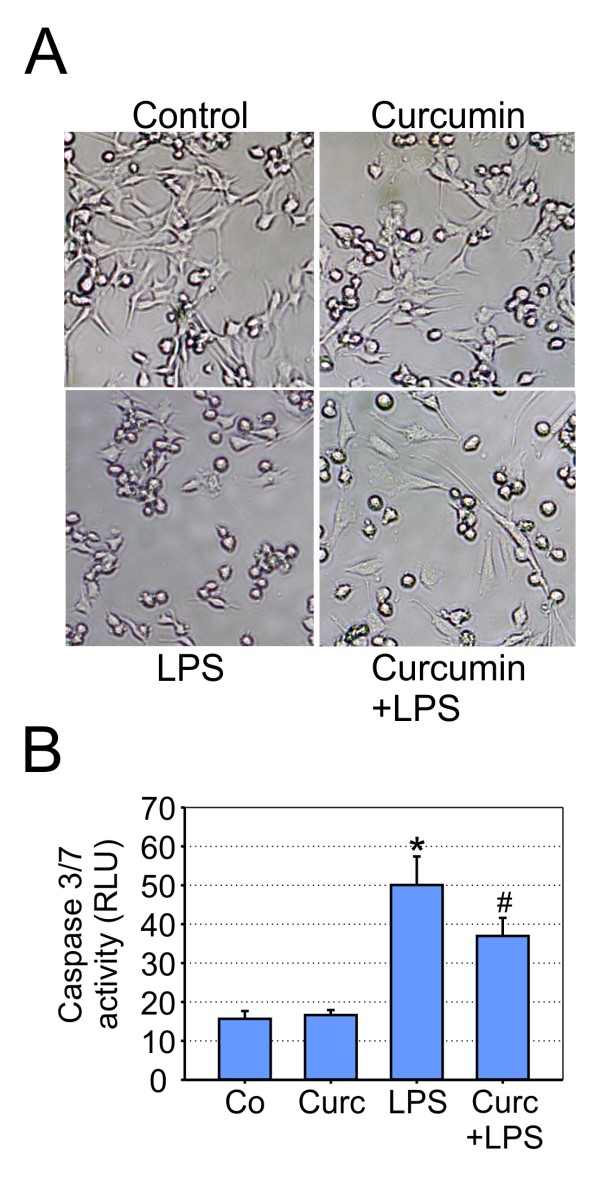
**Curcumin reduces microglial neurotoxicity on photoreceptors**. (A) Phase contrast micrographs showing morphological changes of 661W photoreceptor cell cultures treated with conditioned media from BV-2 cells for 48 hours. The supernatant from control-stimulated, 20 μM curcumin-treated, 100 ng/ml LPS-treated, or 20 μM curcumin + 100 ng/ml LPS-treated cells was added to 661W photoreceptor cells, respectively. The micrographs shown are from one representative experiment out of three independent experiments with the same tendencies. (B) Apoptosis-related caspase 3/7 activation in 661W photoreceptor cells incubated with conditioned media from control-stimulated, 20 μM curcumin-treated, 100 ng/ml LPS-treated, or 20 μM curcumin + 100 ng/ml LPS-treated BV-2 cells. Results are calculated from three independent experiments performed in duplicate measurements. * p ≤ 0.05 for LPS vs. control and # p ≤ 0.05 for curcumin + LPS vs. LPS, respectively, Mann-Whitney Rank Sum test. RLU, relative luciferase units.

To test whether the transcriptomic changes in curcumin-stimulated cells influence microglial neurotoxicity, 661W photoreceptor cells were incubated with conditioned medium from BV-2 cells. 661W is a retinoblastoma-derived cell line, which represents an established model to study microglial neurotoxicity in the special context of retinal degeneration [30,31,35]. 661W cells were incubated for 48 h with culture supernatants from unstimulated, curcumin-, LPS- or LPS + curcumin-treated BV-2 cells and 661W photoreceptor cell morphology was assessed by phase contrast microscopy. 661W cells in their own medium grew in a spindle-like shape with only few rounded apoptotic cells (Figure 5A). Conditioned media from control- or curcumin-treated microglial cells did not affect this morphology (Figure 5A). In contrast, 661W cells incubated with LPS-stimulated BV-2 supernatant appeared apoptotic, leading to larger cell-free areas in the culture (Figure 5A). When conditioned media from LPS + curcumin-stimulated BV-2 cells was used, a nearly normal 661W cell morphology was retained (Figure 5A). Direct incubation of 661W cells with curcumin, LPS, or both had no effects on the cell cultures (data not shown), demonstrating that the observed changes in 661W cell characteristics stem from secreted microglial compounds.

To corroborate these morphological findings with further functional data, we analyzed the influence of microglia-derived products on caspase-related apoptotic cell death. 661W cells cultured with supernatants from LPS-stimulated BV-2 cells showed a significant induction of caspase 3/7 activity (Figure 5B). When using conditioned media from microglial cells co-treated with LPS + curcumin, 661W apoptosis was still present but was significantly diminished (Figure 5B). These data clearly implicate that curcumin may limit the production of pro-apoptotic compounds in activated microglial cells or even promote the release of neurotrophic factors.

## Discussion

Oxidative stress and neuroinflammation are major factors in the pathogenesis of neurodegenerative disorders [36]. Therefore, antioxidant and anti-inflammatory compounds like curcumin may be treatment options for this group of diseases [37]. However, only few experimental data are available that report on curcumin-triggered transcriptional mechanisms and direct signaling targets in microglia.

Our transcriptomic analysis in BV-2 cells sheds some light on target genes and potential signaling mechanisms. We identified a prominent transcriptional response of resting as well as LPS-activated microglial cells after curcumin treatment. Distinct gene clusters were detected that reflect up-regulated and suppressed transcripts in both microglial phenotypes. We identifed and validated six genes that were constistently induced in resting as well as activated BV-2 cells that have not been described as curcumin targets before. Among these, four curcumin target genes are related to cell migration. Netrin G1 is a lipid-anchored protein that is structurally related to the netrin family of axon guidance molecules [38]. It regulates synaptic interactions between neurons by binding to transmembrane netrin G ligands [39]. Interestingly, the related Netrin 1 molecule is a broad inhibitor of leukocyte chemotaxis [40] and Netrin G1 may have a similar function in microglia. The adhesion molecule PECAM1 is also directly involved in monocyte/macrophage migration [41]. Another migration-related gene induced by curcumin is Plasma cell endoplasmic reticulum protein 1. PERP 1 is a molecular chaperone required for proper folding and secretion of immunoglobulins in B-cells [42,43]. Related to our study, a recent report linked PERP 1 (alias MZB1) to calcium signaling, activation of integrins and cell adhesion [44]. Expression of the Notch-ligand Delta-like 1 has been demonstrated in BV-2 cells and primary rat brain microglial cells, where Notch-1 signaling negatively regulates TNF release [45]. Our data show that basal Dll1 expression in resting microglial cells can be potently induced by curcumin, which could potentially trigger Notch-signaling to prevent migration associated with pro-inflammatory priming of BV-2 cells.

These transcriptomic data of curcumin-treatment promoted us to analyze its effects on microglial motility. Both types of assays, the wound healing assays and the transwell migration experiments, showed that BV-2 cell migration was significantly inhibited by 20 μM curcumin over a period of 12 hours to 24 hours. These findings are in good agreement with papers reporting reduced migration of tumor cells, endothelial cells, and dendritic cells after treatment with comparable doses of cucumin [46-48]. In the homeostatic state, microglia constantly scan their environment with their long protrusions without movement of the somata [5]. In contrast, migration of microglial cells is a hallmark of pro-inflammatory and chronic activation during early phases of neurodegeneration. Thus, curcumin may support the homeostatic state of microglia and prevent their early and excessive transformation into migrating phagocytes.

It is well known that curcumin broadly inhibits pro-inflammatory gene expression by targeting different signal pathways and transcriptional regulators including NFkB, AP1, EGR1, and STAT3 [49]. Our microarray data corroborate these findings especially in LPS-activated BV-2 cells by showing curcumin-triggered suppression of Ptgs2, Ccl2, Il6, and Nos2, which are NFkB, AP1, and STAT3 target genes [21]. Moreover, the curcumin-regulated transcriptomic profiles revealed lower gene expression of toll-like receptor 2 in resting microglia and complement factor 3 in activated cells. These two factors broadly support the conversion of microglial cells to the pro-inflammatory state [50,51] and hence curcumin signaling may abrogate both pathways. Our data also showed diminished mRNA expression of the transcription factors Egr2 and Stat1 following curcumin-treatment. This indicates that curcumin may further dampen microglial activation by interfering with two other key transcription factors expressed in activated microglial cells. In addition to its inhibitory effects on pro-inflammatory signaling, two well known anti-inflammatory molecules, PPARα and IL4, were significantly induced by curcumin. PPARα and IL4 both specifically inhibit pro-inflammatory activation of microglial cells [52,53] and some of the immune-dampening effects of curcumin may be mediated via this signaling axis.

The cell culture experiments with conditioned media from BV-2 cells showed that curcumin significantly reduced LPS-triggered microglial neurotoxicity on 661W photoreceptor cells. We hypothesize that the strong suppression of LPS-induced Nos2 transcription by curcumin is a major pathway responsible for this phenomenon. In this context, Mandal *et al*. have recently demonstrated that curcumin protects 661W cells from hydrogen peroxide-induced cell death [29]. This effect is very likely mediated by the antioxidant and radical-scavenging capacity of curcumin. In a model of light-induced retinal degeneration, curcumin also suppressed inflammatory marker expression *in vivo *[29], which could be potentially mediated by its attenuating effect on retinal microglia.

## Conclusions

We have shown that curcumin triggered global changes in the transcriptome of resting and LPS-activated microglial cells. In addition to its known function in blocking pro-inflammatory gene expression via interference with NFkB signaling, curcumin induced novel anti-inflammatory targets in microglia. Curcumin also significantly inhibited microglial migration and cytotoxicity, which are key features of neuroinflammation. Our publicily avialable dataset provides a basis to understand the pleiotropic beneficial effects of curcumin on microglia as key innate immune cells of the nervous system. Moreover, the results of this study also underscore the importance of curcumin as a promising dietary compound for the treatment of various neurodegenerative disorders associated with inflammation.

## Competing interests

The authors declare that they have no competing interests.

## Authors' contributions

MK, EL, and YW carried out cell culture stimulations and qRT-PCR experiments. MK and EL analyzed qRT-PCR and functional data. CM performed microarray analysis. AA performed scratch assays. MM critically read and corrected the paper. TL designed the study, obtained funding, carried out biostatistical analyses of microarrays and wrote the manuscript. All authors read and approved the final manuscript.

## References

[B1] Hanisch UK Kettenmann H Microglia: active sensor and versatile effector cells in the normal and pathologic brain Nat Neurosci 2007 10 1387 1394 10.1038/nn1997 17965659

[B2] Streit WJ Microglia as neuroprotective, immunocompetent cells of the CNS Glia 2002 40 133 139 10.1002/glia.10154 12379901

[B3] Giulian D Li J Bartel S Broker J Li X Kirkpatrick JB Cell surface morphology identifies microglia as a distinct class of mononuclear phagocyte J Neurosci 1995 15 7712 7726 7472522 10.1523/JNEUROSCI.15-11-07712.1995 PMC6578049

[B4] Davalos D Grutzendler J Yang G Kim JV Zuo Y Jung S Littman DR Dustin ML Gan WB ATP mediates rapid microglial response to local brain injury in vivo Nat Neurosci 2005 8 752 758 10.1038/nn1472 15895084

[B5] Nimmerjahn A Kirchhoff F Helmchen F Resting microglial cells are highly dynamic surveillants of brain parenchyma in vivo Science 2005 308 1314 1318 10.1126/science.1110647 15831717

[B6] Broderick C Hoek RM Forrester JV Liversidge J Sedgwick JD Dick AD Constitutive retinal CD200 expression regulates resident microglia and activation state of inflammatory cells during experimental autoimmune uveoretinitis Am J Pathol 2002 161 1669 1677 10.1016/S0002-9440(10)64444-6 12414514 PMC1850781

[B7] Cardona AE Pioro EP Sasse ME Kostenko V Cardona SM Dijkstra IM Huang D Kidd G Dombrowski S Dutta R Control of microglial neurotoxicity by the fractalkine receptor NatNeurosci 2006 9 917 924 10.1038/nn1715 16732273

[B8] Dick AD Carter D Robertson M Broderick C Hughes E Forrester JV Liversidge J Control of myeloid activity during retinal inflammation JLeukocBiol 2003 74 161 166 10.1189/jlb.1102535 12885931

[B9] Haynes SE Hollopeter G Yang G Kurpius D Dailey ME Gan WB Julius D The P2Y12 receptor regulates microglial activation by extracellular nucleotides NatNeurosci 2006 9 1512 1519 10.1038/nn1805 17115040

[B10] Ransohoff RM Perry VH Microglial physiology: unique stimuli, specialized responses Annu Rev Immunol 2009 27 119 145 10.1146/annurev.immunol.021908.132528 19302036

[B11] El Khoury J Luster AD Mechanisms of microglia accumulation in Alzheimer's disease: therapeutic implications Trends Pharmacol Sci 2008 29 626 632 10.1016/j.tips.2008.08.004 18835047

[B12] Orr CF Rowe DB Halliday GM An inflammatory review of Parkinson's disease Prog Neurobiol 2002 68 325 340 10.1016/S0301-0082(02)00127-2 12531233

[B13] Sargsyan SA Monk PN Shaw PJ Microglia as potential contributors to motor neuron injury in amyotrophic lateral sclerosis Glia 2005 51 241 253 10.1002/glia.20210 15846792

[B14] Raivich G Banati R Brain microglia and blood-derived macrophages: molecular profiles and functional roles in multiple sclerosis and animal models of autoimmune demyelinating disease Brain Res Brain Res Rev 2004 46 261 281 15571769 10.1016/j.brainresrev.2004.06.006

[B15] Langmann T Microglia activation in retinal degeneration JLeukocBiol 2007 81 1345 1351 10.1189/jlb.0207114 17405851

[B16] Schuetz E Thanos S Microglia-targeted pharmacotherapy in retinal neurodegenerative diseases CurrDrug Targets 2004 5 619 627 10.2174/1389450043345164 15473251

[B17] Schwartz M Modulating the immune system: a vaccine for glaucoma? Can J Ophthalmol 2007 42 439 441 10.3129/i07-050 17508041

[B18] Zhang Z Zhang ZY Schluesener HJ Compound A, a plant origin ligand of glucocorticoid receptors, increases regulatory T cells and M2 macrophages to attenuate experimental autoimmune neuritis with reduced side effects J Immunol 2009 183 3081 3091 10.4049/jimmunol.0901088 19675162

[B19] Jang S Johnson RW Can consuming flavonoids restore old microglia to their youthful state? Nutr Rev 2010 68 719 728 10.1111/j.1753-4887.2010.00336.x 21091915 PMC3058829

[B20] Ammon HP Wahl MA Pharmacology of Curcuma longa Planta Med 1991 57 1 7 10.1055/s-2006-960004 2062949

[B21] Maheshwari RK Singh AK Gaddipati J Srimal RC Multiple biological activities of curcumin: a short review Life Sci 2006 78 2081 2087 10.1016/j.lfs.2005.12.007 16413584

[B22] Jagetia GC Aggarwal BB "Spicing up" of the immune system by curcumin J Clin Immunol 2007 27 19 35 10.1007/s10875-006-9066-7 17211725

[B23] Jung KK Lee HS Cho JY Shin WC Rhee MH Kim TG Kang JH Kim SH Hong S Kang SY Inhibitory effect of curcumin on nitric oxide production from lipopolysaccharide-activated primary microglia Life Sci 2006 79 2022 2031 10.1016/j.lfs.2006.06.048 16934299

[B24] Jin CY Lee JD Park C Choi YH Kim GY Curcumin attenuates the release of pro-inflammatory cytokines in lipopolysaccharide-stimulated BV2 microglia Acta Pharmacol Sin 2007 28 1645 1651 10.1111/j.1745-7254.2007.00651.x 17883952

[B25] Kang G Kong PJ Yuh YJ Lim SY Yim SV Chun W Kim SS Curcumin suppresses lipopolysaccharide-induced cyclooxygenase-2 expression by inhibiting activator protein 1 and nuclear factor kappab bindings in BV2 microglial cells J Pharmacol Sci 2004 94 325 328 10.1254/jphs.94.325 15037818

[B26] Kim HY Park EJ Joe EH Jou I Curcumin suppresses Janus kinase-STAT inflammatory signaling through activation of Src homology 2 domain-containing tyrosine phosphatase 2 in brain microglia J Immunol 2003 171 6072 6079 14634121 10.4049/jimmunol.171.11.6072

[B27] He LF Chen HJ Qian LH Chen GY Buzby JS Curcumin protects pre-oligodendrocytes from activated microglia in vitro and in vivo Brain Res 2010 1339 60 69 20403340 10.1016/j.brainres.2010.04.014

[B28] Yang S Zhang D Yang Z Hu X Qian S Liu J Wilson B Block M Hong JS Curcumin protects dopaminergic neuron against LPS induced neurotoxicity in primary rat neuron/glia culture Neurochem Res 2008 33 2044 2053 10.1007/s11064-008-9675-z 18368483

[B29] Mandal MN Patlolla JM Zheng L Agbaga MP Tran JT Wicker L Kasus-Jacobi A Elliott MH Rao CV Anderson RE Curcumin protects retinal cells from light-and oxidant stress-induced cell death Free Radic Biol Med 2009 46 672 679 10.1016/j.freeradbiomed.2008.12.006 19121385 PMC2810836

[B30] Dirscherl K Karlstetter M Ebert S Kraus D Hlawatsch J Walczak Y Moehle C Fuchshofer R Langmann T Luteolin triggers global changes in the microglial transcriptome leading to a unique anti-inflammatory and neuroprotective phenotype J Neuroinflammation 2010 7 3 10.1186/1742-2094-7-3 20074346 PMC2819254

[B31] Ebert S Schoeberl T Walczak Y Stoecker K Stempfl T Moehle C Weber BH Langmann T Chondroitin sulfate disaccharide stimulates microglia to adopt a novel regulatory phenotype J Leukoc Biol 2008 84 736 740 10.1189/jlb.0208138 18550791

[B32] Brazma A Hingamp P Quackenbush J Sherlock G Spellman P Stoeckert C Aach J Ansorge W Ball CA Causton HC Minimum information about a microarray experiment (MIAME)-toward standards for microarray data NatGenet 2001 29 365 371 10.1038/ng1201-365 11726920

[B33] Eichler GS Huang S Ingber DE Gene Expression Dynamics Inspector (GEDI): for integrative analysis of expression profiles Bioinformatics 2003 19 2321 2322 10.1093/bioinformatics/btg307 14630665

[B34] Weigelt K Lichtinger M Rehli M Langmann T Transcriptomic profiling identifies a PU.1 regulatory network in macrophages Biochem Biophys Res Commun 2009 380 308 312 10.1016/j.bbrc.2009.01.067 19167354

[B35] Al-Ubaidi MR Font RL Quiambao AB Keener MJ Liou GI Overbeek PA Baehr W Bilateral retinal and brain tumors in transgenic mice expressing simian virus 40 large T antigen under control of the human interphotoreceptor retinoid-binding protein promoter JCell Biol 1992 119 1681 1687 10.1083/jcb.119.6.1681 PMC2289740 1334963

[B36] Hirsch EC Hunot S Neuroinflammation in Parkinson's disease: a target for neuroprotection? Lancet Neurol 2009 8 382 397 10.1016/S1474-4422(09)70062-6 19296921

[B37] Ray B Lahiri DK Neuroinflammation in Alzheimer's disease: different molecular targets and potential therapeutic agents including curcumin Curr Opin Pharmacol 2009 9 434 444 10.1016/j.coph.2009.06.012 19656726

[B38] Lin JC Ho WH Gurney A Rosenthal A The netrin-G1 ligand NGL-1 promotes the outgrowth of thalamocortical axons Nat Neurosci 2003 6 1270 1276 10.1038/nn1148 14595443

[B39] Woo J Kwon SK Kim E The NGL family of leucine-rich repeat-containing synaptic adhesion molecules Mol Cell Neurosci 2009 42 1 10 10.1016/j.mcn.2009.05.008 19467332

[B40] Ly NP Komatsuzaki K Fraser IP Tseng AA Prodhan P Moore KJ Kinane TB Netrin-1 inhibits leukocyte migration in vitro and in vivo Proc Natl Acad Sci USA 2005 102 14729 14734 10.1073/pnas.0506233102 16203981 PMC1253572

[B41] Jackson DE The unfolding tale of PECAM-1 FEBS Lett 2003 540 7 14 10.1016/S0014-5793(03)00224-2 12681475

[B42] Shimizu Y Meunier L Hendershot LM pERp1 is significantly up-regulated during plasma cell differentiation and contributes to the oxidative folding of immunoglobulin Proc Natl Acad Sci USA 2009 106 17013 17018 10.1073/pnas.0811591106 19805157 PMC2761359

[B43] van Anken E Pena F Hafkemeijer N Christis C Romijn EP Grauschopf U Oorschot VM Pertel T Engels S Ora A Efficient IgM assembly and secretion require the plasma cell induced endoplasmic reticulum protein pERp1 Proc Natl Acad Sci USA 2009 106 17019 17024 10.1073/pnas.0903036106 19805154 PMC2761347

[B44] Flach H Rosenbaum M Duchniewicz M Kim S Zhang SL Cahalan MD Mittler G Grosschedl R Mzb1 protein regulates calcium homeostasis, antibody secretion, and integrin activation in innate-like B cells Immunity 33 723 735 10.1016/j.immuni.2010.11.013 PMC3125521 21093319

[B45] Cao Q Lu J Kaur C Sivakumar V Li F Cheah PS Dheen ST Ling EA Expression of Notch-1 receptor and its ligands Jagged-1 and Delta-1 in amoeboid microglia in postnatal rat brain and murine BV-2 cells Glia 2008 56 1224 1237 10.1002/glia.20692 18449946

[B46] Senft C Polacin M Priester M Seifert V Kogel D Weissenberger J The nontoxic natural compound Curcumin exerts anti-proliferative, anti-migratory, and anti-invasive properties against malignant gliomas BMC Cancer 2010 10 491 10.1186/1471-2407-10-491 20840775 PMC2949804

[B47] Sameermahmood Z Balasubramanyam M Saravanan T Rema M Curcumin modulates SDF-1alpha/CXCR4-induced migration of human retinal endothelial cells (HRECs) Invest Ophthalmol Vis Sci 2008 49 3305 3311 10.1167/iovs.07-0456 18660423

[B48] Shirley SA Montpetit AJ Lockey RF Mohapatra SS Curcumin prevents human dendritic cell response to immune stimulants Biochem Biophys Res Commun 2008 374 431 436 10.1016/j.bbrc.2008.07.051 18639521 PMC3319308

[B49] Shishodia S Singh T Chaturvedi MM Modulation of transcription factors by curcumin Adv Exp Med Biol 2007 595 127 148 10.1007/978-0-387-46401-5_4 17569208

[B50] Lin HY Tang CH Chen YH Wei IH Chen JH Lai CH Lu DY Peptidoglycan enhances proinflammatory cytokine expression through the TLR2 receptor, MyD88, phosphatidylinositol 3-kinase/AKT and NF-kappaB pathways in BV-2 microglia Int Immunopharmacol 2010 10 883 891 10.1016/j.intimp.2010.04.026 20451669

[B51] Fan R DeFilippis K Van Nostrand WE Induction of complement proteins in a mouse model for cerebral microvascular A beta deposition J Neuroinflammation 2007 4 22 10.1186/1742-2094-4-22 17877807 PMC2099424

[B52] Xu J Storer PD Chavis JA Racke MK Drew PD Agonists for the peroxisome proliferator-activated receptor-alpha and the retinoid × receptor inhibit inflammatory responses of microglia J Neurosci Res 2005 81 403 411 10.1002/jnr.20518 15968640

[B53] Lyons A McQuillan K Deighan BF O'Reilly JA Downer EJ Murphy AC Watson M Piazza A O'Connell F Griffin R Decreased neuronal CD200 expression in IL-4-deficient mice results in increased neuroinflammation in response to lipopolysaccharide Brain Behav Immun 2009 23 1020 1027 10.1016/j.bbi.2009.05.060 19501645

